# Global Outbreak Alert and Response Network deployments during the COVID-19 pandemic, WHO Western Pacific Region

**DOI:** 10.5365/wpsar.2024.15.5.1060

**Published:** 2024-02-06

**Authors:** Sharon Salmon, Simon Brinkwirth, Gianluca Loi, Jocelyne M Basseal

**Affiliations:** a Global Outbreak Alert and Response Network, Emergency Operations, WHO Health Emergencies Programme, World Health Organization Regional Office for the Western Pacific, Manila, Philippines.; b Indo-Pacific Centre for Health Security, Department of Foreign Affairs and Trade, Canberra, Australia.; c Global Outbreak Alert and Response Network, Department of Alert and Response Coordination, WHO Health Emergencies Programme, World Health Organization, Geneva, Switzerland.; d Postgraduate Training for Applied Epidemiology, Robert Koch Institute, Berlin, Germany.; e European Programme for Intervention Epidemiology Training, European Centre for Disease Prevention and Control, Stockholm, Sweden.; f Sydney Infectious Diseases Institute, Faculty of Medicine and Health, The University of Sydney, Sydney, New South Wales, Australia.

## Abstract

**Problem:**

The Global Outbreak Alert and Response Network (GOARN) has responded to more than 100 outbreaks during the past 23 years. The coronavirus disease (COVID-19) pandemic presented unprecedented operational constraints that challenged GOARN’s core mission to rapidly deploy technical experts from its partners to support national in-country responses to public health emergencies. This paper describes the type and duration of GOARN deployments to and within the World Health Organization’s (WHO’s) Western Pacific Region during the COVID-19 pandemic.

**Context:**

Despite strict border closures and ever-changing vaccination and quarantine requirements, GOARN continued to deploy international technical assistance to strengthen COVID-19 response operations within the Region, as requested.

**Action:**

Data were analysed from the GOARN Knowledge Platform about deployments to and within the Region for responses to the COVID-19 pandemic between 1 January 2020 and 5 May 2023. Data were available about deployment duration, technical role requested, country or area, partner organization and deployed expert’s demographics. Feedback from postdeployment briefings with the experts was collected and thematically analysed to determine ongoing needs and gaps to help improve deployment operations.

**Outcome:**

There were 72 experts deployed on 89 missions through GOARN to 12 countries and areas in the Region, for a total of 4558 field days, to support the response to the COVID-19 pandemic.

**Discussion:**

The volume of requests for assistance from countries and areas in the Region to respond to the COVID-19 pandemic uncovered a deficit in human resources available for domestic response to outbreaks and the reliance on international assistance. Strengthening the in-country capacity of ready-to-respond public health emergency staff is critical to meet the needs for outbreak response. The ongoing demand for technical experts to support national responses means that these lessons may have immediate implications.

## PROBLEM

Requests for deployments made through the Global Outbreak Alert and Response Network (GOARN) continued during the coronavirus disease (COVID-19) pandemic, but these were more challenging to facilitate due to the public health and social measures implemented to control disease transmission. International border closures, restricted and cancelled flights, visa processing delays, and vaccination and quarantine requirements enforced by countries and areas complicated and delayed the rapid deployment of experts through the GOARN mechanism. The increase in requests also highlighted deficits in the availability of local experts with the technical skills required to support outbreak response activities. Therefore, the objective of this paper is to describe, for the first time, the type and duration of GOARN deployments to and within the World Health Organization (WHO) Western Pacific Region during the COVID-19 pandemic, including the issues encountered, and to propose operational improvements.

## CONTEXT

To fulfil its mandate under the International Health Regulations (2005), WHO rapidly and consistently identifies and assesses events of potential international public health concern. Based on this assessment, WHO alerts its Member States about such threats and provides technical assistance to affected countries and areas during their investigation and control efforts, if requested. (*1*) In rare circumstances, the WHO Director-General may declare events to be public health emergencies of international concern. For such events, including the COVID-19 pandemic, WHO establishes an incident management support team at national, regional and global levels. The responsibilities of this team are set out in WHO’s *Emergency response framework* and include coordinating and supporting technical assistance for health operations in countries, and liaising with GOARN and other partners. (*2*)

Established by WHO in April 2000, GOARN is a mechanism for technical partners and networks around the world to coordinate and assist WHO Member States in responding to public health emergencies. (*3*) GOARN’s partners include more than 300 technical institutions, organizations and networks, and they can pool their resources to improve communications and information-sharing about emerging and ongoing public health events, and support capacities for preparedness and rapid outbreak response. GOARN’s partners are drawn from national, regional and global stakeholders and include ministries of health; national public health institutes; medical, surveillance and laboratory networks; United Nations organizations; International Federation of Red Cross and Red Crescent Societies; nongovernmental organizations; academic institutions; and technical networks. Critical to the success of an outbreak response is the engagement of a multidisciplinary pool of trained experts (*4*) who have the skills to gain trust, lead and work with local health authorities and communities. (*5*)

Depending on the magnitude of the event and the national capacity for operational responses, national health authorities may request international technical assistance through the WHO incident management support team. When a request for assistance is initiated, the WHO GOARN Operational Support Team (OST) notifies all GOARN partner institutions through their nominated focal point, detailing the required technical role, including academic qualifications and work experience, the terms of reference, location and minimum deployment duration. Requests for assistance are published via the web-based GOARN Knowledge Platform, (*6*) accessible to focal points at GOARN’s partner organizations.

Focal points at GOARN’s partner organizations assess the capacity of their institution and individual experts to provide the support requested. Offers of assistance are uploaded by partners onto the GOARN Knowledge Platform. The GOARN OST reviews and shares offers that meet all requirements with the WHO regional office or country office, as applicable. Offers are then shared with the national authorities who made the request, and they determine which expert(s) will be selected. The GOARN OST and WHO regional and country offices work together to actualize the deployment. Experts selected for deployment are issued a short-term consultant contract by WHO, without remuneration, which includes basic health insurance, security coverage, travel costs and a daily living allowance.

Since GOARN’s inception in 2000, its partners have completed deployments to more than 117 countries and areas, involving more than 3573 experts with approximately 122 000 field days. GOARN deployments for the response to the COVID-19 pandemic from 1 January 2020 to 5 May 2023 included 249 experts deployed to 46 countries and areas worldwide. (*6*)

### GOARN in the Western Pacific Region

The Western Pacific Region has a population of approximately 1.9 billion people across 37 countries and areas. The Region suffers a disproportionate burden of preventable infectious disease, and the outbreak response capacity is variable. (*7*) Increasing urbanization, faster connectivity, globalization and the impacts of climate change have heightened the ever-present risk of health emergencies and the emergence of new health security threats. (*7*) Since 2000, the Region has experienced outbreaks of diseases such as severe acute respiratory syndrome, avian influenza in humans, Middle East respiratory syndrome, dengue, influenza H1N1 and Zika virus disease. (*7*)

There are 73 GOARN partner organizations located in the Region at universities, colleges, hospitals, public health and technical institutions and networks, and governmental departments and agencies. Since 2000, 299 experts have deployed through GOARN from its partner organizations in the Region to 36 global operations. (*6*)

## ACTION

Data were obtained from the GOARN Knowledge Platform (*6*) about deployments to the Region for the COVID-19 pandemic response from 1 January 2020 to 5 May 2023. Data were available about deployment duration, technical role requested, country or area, partner organization and deployed expert’s demographics. Feedback from postdeployment briefings with the experts was collected and thematically analysed to determine ongoing needs and gaps to help support improved deployment operations.

Between January 2020 and May 2023, 72 experts completed 89 GOARN deployments to 12 countries and areas to assist in responses to the COVID-19 pandemic in the Region. There were 35 deployments in 2020, 30 in 2021 and 24 in 2022. Deployments to the Region represented 36% (89/249) of all global GOARN deployments for the COVID-19 pandemic response.

**Fig. 1:**
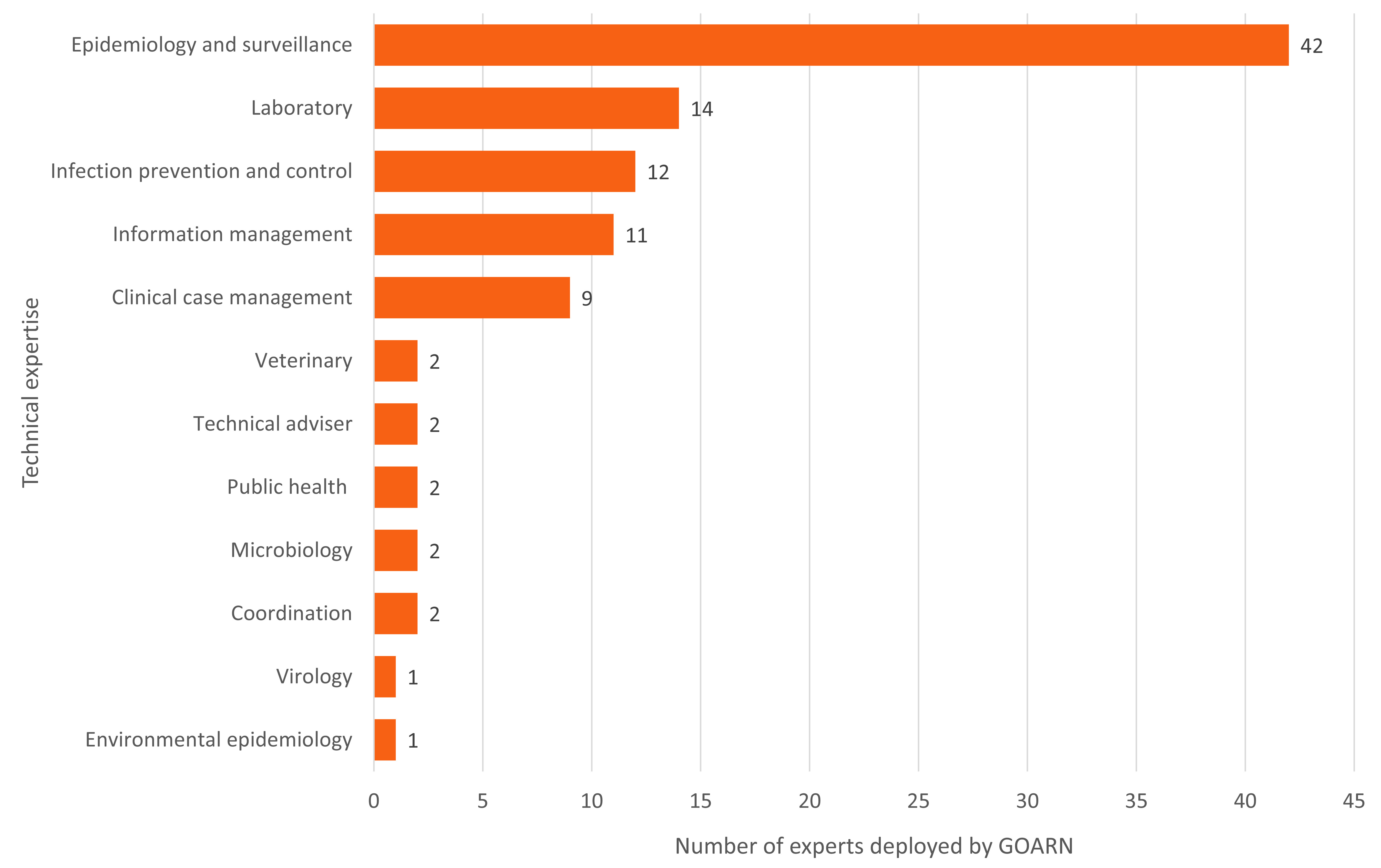
Number of experts deployed by the Global Outbreak Alert and Response Network (GOARN) during the COVID-19 response in the WHO Western Pacific Region, by area of technical expertise, 1 January 2020 to 5 May 2023a

**Fig. 2:**
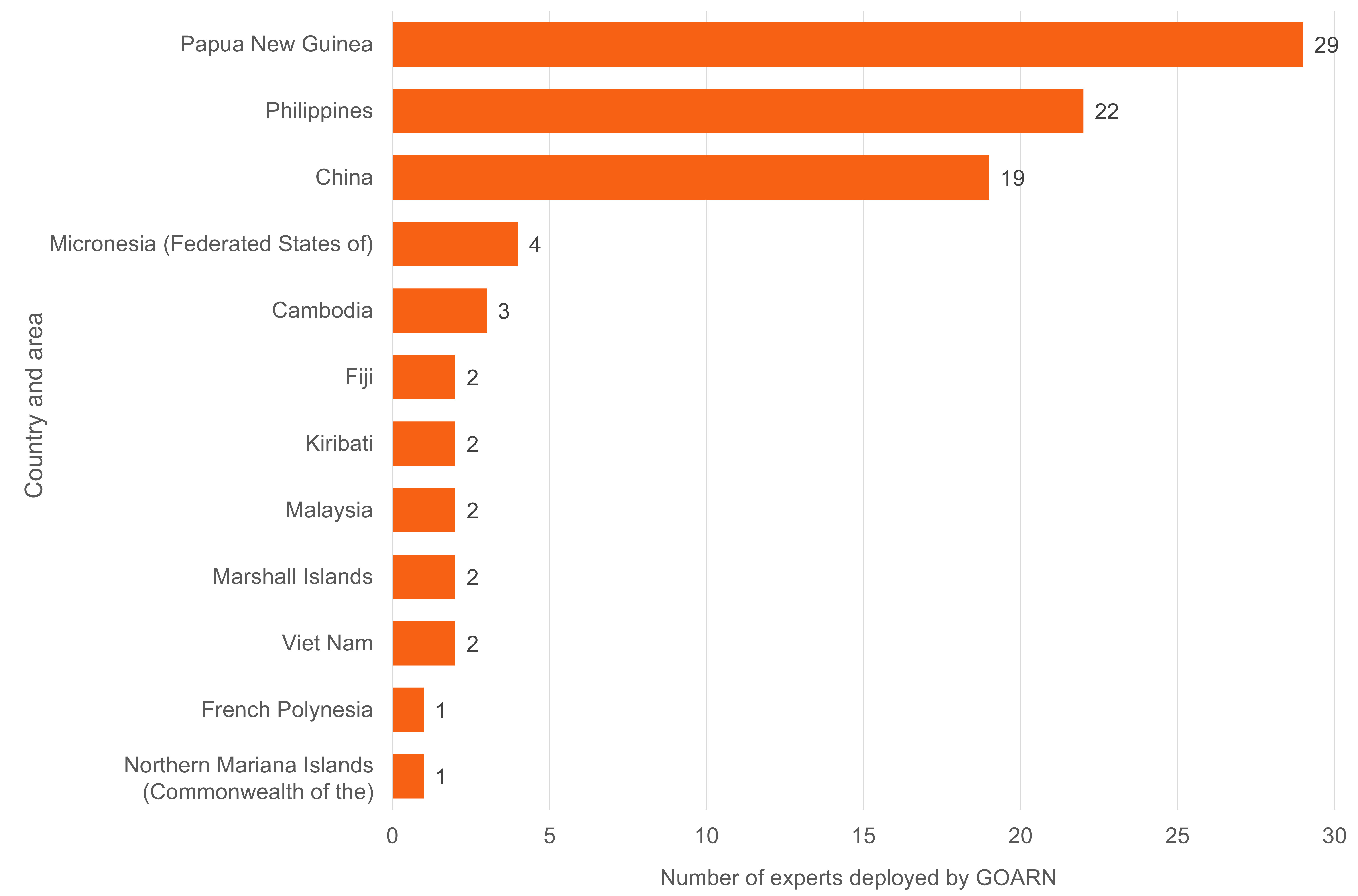
Number of deployments by the Global Outbreak Alert and Response Network (GOARN) during the COVID-19 response in the WHO Western Pacific Region, by location of deployment, 1 January 2020 to 5 May 2023

Experts were deployed from 34 of GOARN’s partners, 16 (47%) from within the Region. The most requested categories of technical expertise were epidemiology and surveillance, data management, laboratory and infection prevention and control. As experts can provide support across more than one area of technical expertise, there were 100 instances of technical expertise provided (**Fig. 1**). Thirty-four of the 72 experts deployed (47%) were female. The country or area with the highest number of deployments was Papua New Guinea (*n* = 29), followed by the Philippines (*n* = 22) and China (*n* = 19) (**Fig. 2**).

[**insert** Figure 1]

[**insert** Figure 2]

**Fig. 3:**
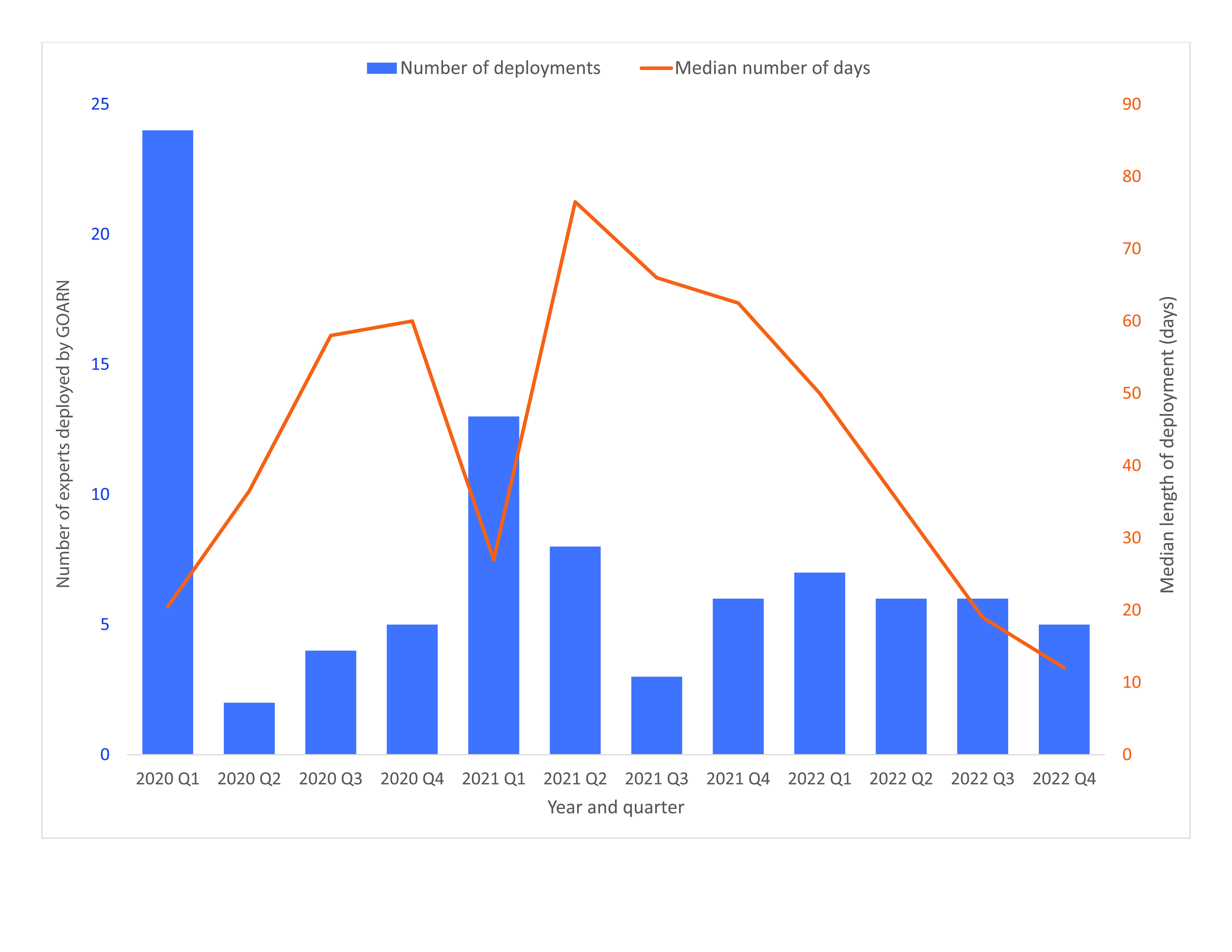
Number of experts deployed by the Global Outbreak Alert and Response Network (GOARN) and median duration of deployment during the COVID-19 response in the WHO Western Pacific Region, by quarter, 1 January 2020 to 5 May 2023

The duration of the deployments ranged from 8 to 139 days (median: 36 days). The longest deployment was to Papua New Guinea for 139 days, followed by the Philippines for 133 days. The highest number of deployments to the Region occurred during the first quarter of 2020 (*n* = 24), at the beginning of the COVID-19 pandemic, and included not only individual deployments but also two team missions to China. The smaller number of deployments during the second quarter of 2020 (*n* = 2) coincided with strict international border closures, travel restrictions and quarantine measures (**Fig. 3**).

[**insert** Figure 3]

The majority of deployments involved individual experts sent to support the COVID-19 response in a single country or area. Deployments to China supported two separate WHO–China joint team missions: the first in February 2020 to conduct a field visit to Wuhan to understand the response to severe acute respiratory syndrome coronavirus 2 (SARS-CoV-2), and the second in January and February 2021 to strengthen scientific cooperation on work to trace the origin of the novel coronavirus. One infection prevention and control expert deployed to the WHO Regional Office for the Western Pacific also provided in-country support for the COVID-19 response to Brunei Darussalam and the Lao People's Democratic Republic during the same mission. With the easing of border restrictions and the increased availability of flights, a team of two experts was deployed in-country to the Federated States of Micronesia and the Marshall Islands, and one senior expert with extensive field experience provided remote assistance to the team.

## OUTCOME

GOARN’s partners demonstrated their capacity to respond internationally despite competing domestic demands for response activities. Effective and safe deployments were possible in the Region even during the extraordinary and unpredictable time of the COVID-19 pandemic. However, there were several challenges in implementing these deployments. Partners’ focal points required more tailored communications during the COVID-19 response to understand that deployments through GOARN were possible during the pandemic. Focal points play a key role in communications and in selecting and uploading offers from experts who are willing and able to deploy. Focal points certify that the experts proposed by their organization have the technical skills and work experience that match a request’s requirements. Knowledge of the deployment process varied between partners, requiring continual communications between the WHO Regional Office and its partners. Institutes with an international mandate were better equipped to propose experts and support them during deployment.

The demand for GOARN deployments during responses to the COVID-19 pandemic created an exceptional workload for administrative personnel. Being able to rapidly process these deployments was a challenge due to uncertainty about international border restrictions, visa application criteria, delays in visa issuance, limited and/or cancelled international flights, changing COVID-19 testing requirements and the sudden onset of sickness or a laboratory result positive for COVID-19 for the selected expert. These issues all contributed to delays in the speed and efficiency of deploying international experts.

As the COVID-19 pandemic evolved into a protracted emergency, longer deployments were requested. GOARN’s usual deployments last 4–8 weeks; however, the median duration of deployments per quarter exceeded this during the majority of the COVID-19 pandemic response (**Fig. 3**). These longer in-country missions improved contextual understanding, continuity and stability and thus allowed for better-tailored response interventions; they also enabled trust to be built with national counterparts despite highly stressful working conditions and demanding circumstances. However, these longer deployments also precluded many experts from participating due to their lack of time amid competing priorities. Senior experts, who would usually be sent to assist in an international response, were unable to deploy during the COVID-19 pandemic due to competing domestic response commitments resulting from the COVID-19 pandemic. This reduced the pool of experts and forced the deployment of some who had only minimal international field experience.

As the COVID-19 pandemic and requests for GOARN deployments continued, the offers of assistance varied in terms of the qualifications and experience of the experts. Many proposed experts did not meet the minimum requirements outlined in the terms of reference, such as having experience working in an international outbreak team. The nomination of unqualified experts may have indicated an eagerness on the part of less experienced staff to gain international outbreak experience during this protracted emergency.

## Discussion

Despite the challenges, GOARN managed to deploy experts in the Region during the response to the COVID-19 pandemic. In preparation for responses to future large outbreaks and emergencies, and to address some of the challenges that arose, the following recommendations to improve the operational deployment process are proposed.

### Improve focal points’ engagement with GOARN

To address inconsistencies in knowledge about GOARN’s processes among partners’ focal points, regular orientation and/or refresher sessions about processes should be offered. These may ensure that focal points have up-to-date knowledge about their roles and responsibilities. Additionally, to support focal points to quickly identify experts who have the required skills and experience, a roster of ready-to-deploy experts could be compiled for their organization to expedite the process, rather than waiting for experts to propose themselves. More frequent and regular bidirectional communication between the GOARN OST, WHO regional offices and focal points could also support the urgent sourcing and deployment of appropriate experts from neighbouring countries within a region.

### Integrate virtual technical support for deployments

To achieve a mission’s objectives and support less experienced responders to operate in highly stressful field conditions, GOARN should consider having senior experts provide virtual or remote support. Such modalities of team deployment could help by expanding the number of emergency public health staff who are available to respond.

### Improve intra-regional deployments of GOARN’s partners

Conducting a gap analysis of GOARN’s partners within the Region, including documenting partners’ capacity to respond, would help with readiness, could potentially expedite deployments and could result in an increase in the number of deployments within the Region. Intra-regional deployments would potentially reduce travel time and improve other administrative arrangements, such as visa entry requirements. Improving intra-regional engagement with partners requires raising awareness of GOARN’s role and the deployment process. Encouraging GOARN’s partners to undertake GOARN trainings and facilitating opportunities for collaboration between partners would also strengthen regional preparedness and response capacities. Further, increasing the number of partners within the Region may better support and complement national in-country rapid response operations, reducing the reliance on having experts from other regions fly in.

The data presented in this report were limited to those reported on the GOARN Knowledge Platform and the challenges identified during semistructured debriefing sessions with experts and WHO. However, the lessons learned provide opportunities to improve GOARN’s deployment process and re-emphasize the critical importance of partnerships in addressing global health security and solidarity. Ensuring further engagement between GOARN and its partners and national authorities may strengthen domestic response capacities; additionally, encouraging partners to review their own capacity and ability to respond to requests for outbreak response assistance may expedite future deployments. Expanding the number of partners within the Region may provide improved technical support for current and inevitable future outbreaks.
